# Associations between the use of aspirin or other antiplatelet drugs and all-cause mortality among patients with COVID-19: A meta-analysis

**DOI:** 10.3389/fphar.2022.989903

**Published:** 2022-10-05

**Authors:** Wanting Su, He Miao, Zhaotian Guo, Qianhui Chen, Tao Huang, Renyu Ding

**Affiliations:** ^1^ Department of Critical Care Medicine, The First Hospital of China Medical University, Shenyang, Liaoning Province, China; ^2^ Department of Epidemiology & Biostatistics, School of Public Health, Peking University, Beijing, China; ^3^ Key Laboratory of Molecular Cardiovascular Sciences (Peking University), Ministry of Education, Beijing, China; ^4^ Center for Intelligent Public Health, Institute for Artificial Intelligence, Peking University, Beijing, China

**Keywords:** aspirin, antiplatelet drug, mortality, COVID-19, meta-analysis

## Abstract

**Introduction:** Whether aspirin or other antiplatelet drugs can reduce mortality among patients with coronavirus disease (COVID-19) remains controversial.

**Methods:** We identified randomized controlled trials, prospective cohort studies, and retrospective studies on associations between aspirin or other antiplatelet drug use and all-cause mortality among patients with COVID-19 in the PubMed database between March 2019 and September 2021. Newcastle–Ottawa Scale and Cochrane Risk of Bias Assessment Tool were used to assess the risk of bias. The I^2^ statistic was used to assess inconsistency among trial results. The summary risk ratio (RR) and odds ratio (OR) were obtained through the meta-analysis.

**Results:** The 34 included studies comprised three randomized controlled trials, 27 retrospective studies, and 4 prospective cohort studies. The retrospective and prospective cohort studies showed low-to-moderate risks of bias per the Newcastle–Ottawa Scale score, while the randomized controlled trials showed low-to-high risks of bias per the Cochrane Risk of Bias Assessment Tool. The randomized controlled trials showed no significant effect of aspirin use on all-cause mortality in patients with COVID-19 {risk ratio (RR), 0.96 [95% confidence interval (CI) 0.90–1.03]}. In retrospective studies, aspirin reduced all-cause mortality in patients with COVID-19 by 20% [odds ratio (OR), 0.80 (95% CI 0.70–0.93)], while other antiplatelet drugs had no significant effects. In prospective cohort studies, aspirin decreased all-cause mortality in patients with COVID-19 by 15% [OR, 0.85 (95% CI 0.80–0.90)].

**Conclusion:** The administration of aspirin may reduce all-cause mortality in patients with COVID-19.

## 1 Introduction

Coronavirus disease (COVID-19) is associated with a state of prothrombosis that leads to adverse clinical outcomes (Oudkerk et al., 2020; Lopes et al., 2021). Infection by severe acute respiratory syndrome coronavirus 2 (SARS-CoV-2) induces platelet activation and platelet-mediated immune inflammation, which also contributes to thrombosis in patients with COVID-19 (Mei et al., 2020). Meizlish et al. (2021) first reported that intermediate doses of anticoagulants and aspirin were associated with a lower cumulative incidence of in-hospital death in patients with COVID-19. In addition to clopidogrel, dipyridamole, and P2Y12 inhibitors, (Mega and Simon, 2015), aspirin is a powerful antiplatelet drug (Schrör, 1997) that plays an important role in anti-thrombosis, anti-inflammation, and antiviral treatments (Bianconi et al., 2020; Simon et al., 2020; Tantry et al., 2021; Connors and Ridker, 2022).

To our knowledge, published meta-analyses on this topic have only included observational studies, with controversial results (Kow and Hasan, 2021; Martha et al., 2021; Salah and Mehta, 2021; Srivastava and Kumar, 2021; Wang et al., 2021). As of July 2022, a comprehensive systematic search of ClinicalTrials.gov showed 24 registered trials and published results from four randomized controlled trials. To further explore the relationship between aspirin or other antiplatelet drugs and all-cause mortality in patients with COVID-19, we conducted a meta-analysis of randomized controlled trials, prospective cohort studies, and retrospective studies.

This meta-analysis aimed to assess the association between aspirin or other antiplatelet drugs and all-cause mortality in patients with COVID-19.

## 2 Methods

This meta-analysis adhered to the Preferred Reporting Items for Systematic Reviews and Meta-analyses (PRISMA) reporting guidelines.

### 2.1 Search strategy

Relevant articles were searched in the PubMed database from the database inception to July 2022 using the keywords: “COVID-19” and “aspirin”, as well as “COVID-19” and “antiplatelet drugs.”

### 2.2 Inclusion and exclusion criteria

Studies were included according to the inclusion and exclusion criteria by screening the titles and abstracts of the articles to assess their eligibility. Disagreements were resolved through discussion.

The inclusion criteria were: 1) randomized controlled trials, prospective cohort studies, or retrospective studies; 2) patients with confirmed COVID-19; 3) an exposure group administered aspirin or other antiplatelet drugs and a control group receiving usual care or placebo; 4) an outcome of mortality; and 5) reported mortality among patients who received aspirin or other antiplatelet drugs or mortality could be calculated or compared to that of the control group. All other studies were excluded from the analysis. No language restrictions were imposed.

### 2.3 Outcomes

The primary outcome was all-cause mortality, as reported directly in the study or calculated from data included in the paper. The secondary outcomes were serious adverse events, including bleeding and thrombotic events.

### 2.4 Data extraction

Data were abstracted by four authors (WS, HM, ZG, and QC) from 34 studies, (Abu-Jamous et al., 2020; Alamdari et al., 2020; Giacomelli et al., 2020; Lodigiani et al., 2020; Russo et al., 2020; Sahai et al., 2020; Tremblay et al., 2020; Chow et al., 2021a; Chow et al., 2021b; Connors et al., 2021; Fröhlich et al., 2021; Haji Aghajani et al., 2021; Ho et al., 2021; Kim et al., 2021; Liu et al., 2021; Meizlish et al., 2021; Merzon et al., 2021; Mura et al., 2021; Osborne et al., 2021; Pan et al., 2021; Sisinni et al., 2021; Son et al., 2021; Xiang et al., 2021; Yuan et al., 2021; Zhao et al., 2021; Zhou et al., 2021; Al Harthi et al., 2022; Santoro et al., 2022a; Chow et al., 2022; Formiga et al., 2022; Gogtay et al., 2022; RECOVERY Collaborative GroupAbbas et al., 2022; REMAP-CAP Writing Committee for the REMAP-CAP InvestigatorsBradbury et al., 2022; Sullerot et al., 2022), including three randomized controlled trials, (Connors et al., 2021; RECOVERY Collaborative GroupAbbas et al., 2022; REMAP-CAP Writing Committee for the REMAP-CAP InvestigatorsBradbury et al., 2022), 27 retrospective studies, (Abu-Jamous et al., 2020; Alamdari et al., 2020; Lodigiani et al., 2020; Russo et al., 2020; Sahai et al., 2020; Tremblay et al., 2020; Chow et al., 2021a; Chow et al., 2021b; Fröhlich et al., 2021; Haji Aghajani et al., 2021; Ho et al., 2021; Kim et al., 2021; Liu et al., 2021; Meizlish et al., 2021; Merzon et al., 2021; Mura et al., 2021; Osborne et al., 2021; Pan et al., 2021; Sisinni et al., 2021; Son et al., 2021; Yuan et al., 2021; Zhao et al., 2021; Zhou et al., 2021; Al Harthi et al., 2022; Formiga et al., 2022; Gogtay et al., 2022; Sullerot et al., 2022), and four prospective cohort studies (Giacomelli et al., 2020; Xiang et al., 2021; Santoro et al., 2022a; Chow et al., 2022). The extracted data are shown in Supplementary Tables S1–S3.

### 2.5 Quality assessment

The risks of bias in retrospective and prospective cohort studies were assessed using the Newcastle Ottawa Scale (NOS) (Wells et al., 2000), which was also used for the quality assessment of all included retrospective and prospective cohort studies. The risk of bias in the randomized controlled trials was assessed using the Cochrane Risk of Bias Assessment Tool (Sterne et al., 2019).

### 2.6 Data analysis

STATA 16.0 was used to perform the meta-analysis. The risk ratios (RRs) for randomized controlled trials and odds ratios (ORs) for retrospective and prospective cohort studies with their corresponding 95% confidence intervals (95% CIs) were calculated. Owing to the low heterogeneity (I^2^ < 25%), a fixed-effects model was used for the randomized controlled trials with aspirin as an intervention. The random-effects model was used for analyses of retrospective and prospective cohort studies with aspirin or other antiplatelet drugs as interventions. The heterogeneity among the studies was evaluated using the I^2^ statistic, with I^2^ > 25% indicating substantial heterogeneity. Cochran’s Q statistic was used to derive the precise *p*-values for heterogeneity.

Subgroup analysis was performed to assess the associations between aspirin and all-cause mortality at different time points of administration (before or after admission). The associations in the subgroups were compared by calculating the ORs. ORs further from 1 indicated a greater difference between the estimated associations in the two subgroups.

## 3 Results

### 3.1 Study characteristics

**FIGURE 1:**
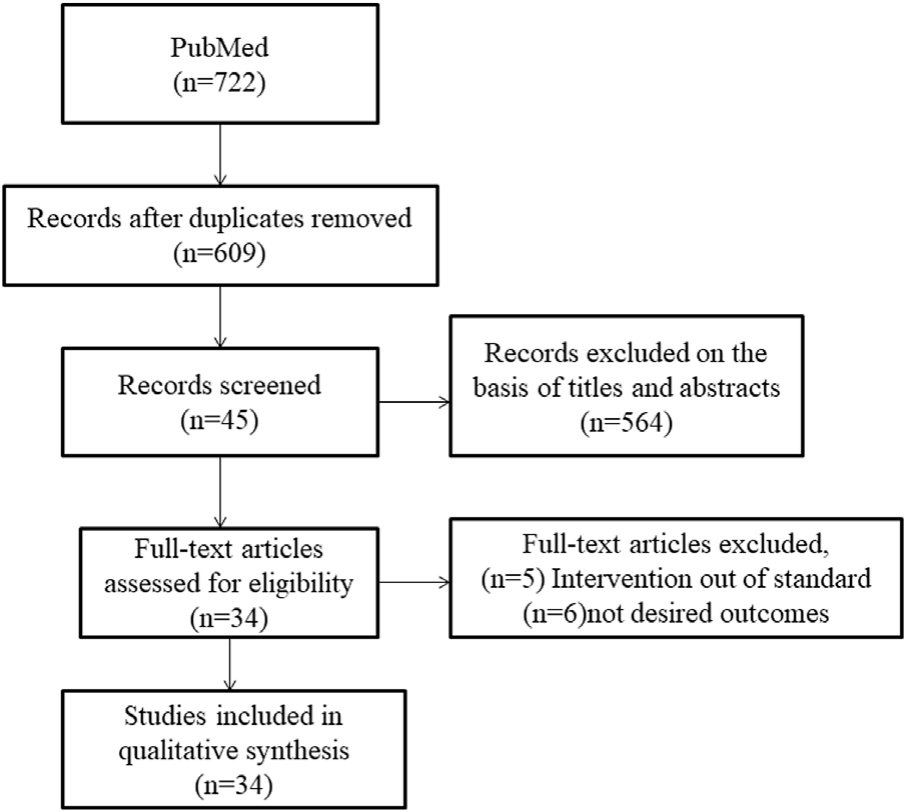
Study selection flowchart.

The initial search of PubMed from its inception through July 2022 revealed a total of 722 studies. A review of the titles and abstracts removed 113 duplicate articles and excluded an additional 564 articles. Finally, the full texts of 45 articles were downloaded and an additional 11 articles were excluded. Four studies (a randomized controlled trial, a prospective cohort study, and two retrospective studies) were excluded as the studies mainly aimed at assessing the effect of anticoagulants, not antiplatelet drugs, on the prognosis of patients with COVID-19 (Viecca et al., 2020; Matli et al., 2021; Berger et al., 2022; Santoro et al., 2022b). One study was excluded as the interventions did not meet the inclusion criteria (Kevorkian et al., 2021). Six studies were excluded as the outcomes were not of interest for our analysis (Argenziano et al., 2020; Dreher et al., 2020; Middeldorp et al., 2020; Regina et al., 2020; Sivaloganathan et al., 2020; Abdelwahab et al., 2021). Finally, the quantitative synthesis comprised 34 articles, including three randomized controlled trials and 17,225 patients with COVID-19, 27 retrospective studies and 96,552 patients, and four prospective cohort studies and 120,019 patients (Figure 1). Among the randomized controlled trials, the sample size of the RECOVERY study was large with a weight of 86.47% (RECOVERY Collaborative GroupAbbas et al., 2022). One retrospective study with enough patients was only applicable to a specific group because it included a homogenous sample from the Veterans Health Administration (Osborne et al., 2021). The study characteristics are summarized in Supplementary Tables 1–3.

### 3.2 Association between aspirin or other antiplatelet drug use and all-cause mortality

**FIGURE 2:**
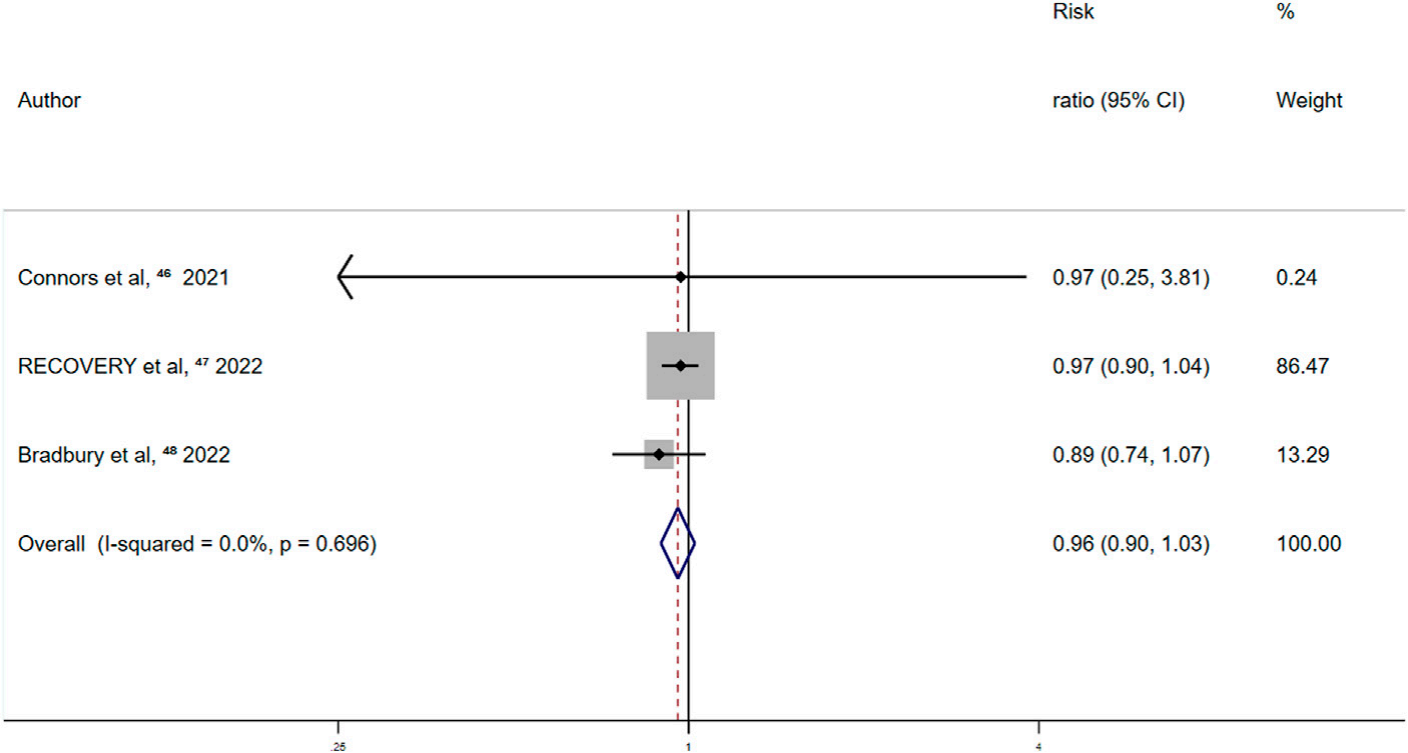
Summary hazard ratio of all-cause mortality for aspirin use in randomized controlled trials.

Among randomized controlled trials exploring the association between aspirin and all-cause mortality, 1,387 (17.2%) and 1,470 (17.9%) deaths were reported in the aspirin and control groups, respectively. With a summary fixed-effects RR of 0.96 (95% CI 0.90–1.03; *p* = 0.222), no between-study heterogeneity was detected (I^2^ = 0.0%; *p* = 0.696) (Figure 2). Thus, aspirin use showed no significant effect on all-cause mortality in patients with COVID-19.

The randomized controlled trial on the association between P2Y12 inhibitors and all-cause mortality reported 127 deaths among 448 patients taking P2Y12 inhibitors and 167 deaths among 521 patients receiving usual care, with an RR of 0.88 (95% CI 0.73–1.07). No significant effect was observed between P2Y12 inhibitor use and all-cause mortality among patients with COVID-19.

One prospective cohort study on the association between aspirin use and all-cause mortality reported 1,410 deaths among 13,795 patients taking aspirin and 11,577 deaths among 98,275 patients receiving usual care, with an OR of 0.85 (95% CI 0.80–0.90). A 15% reduction in all-cause mortality was observed after aspirin use among patients with COVID-19.

**FIGURE 3:**
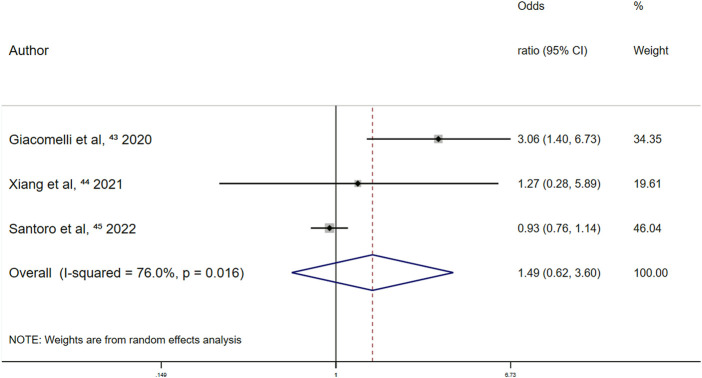
Summary odds ratio of all-cause mortality for other antiplatelet drugs use in prospective cohort studies.

Prospective cohort studies showed no significant effect between the use of other antiplatelet drugs and all-cause mortality among patients with COVID-19 [OR, 1.49 (95% CI 0.62–3.60); *p* = 0.375] (Figure 3).

**FIGURE 4:**
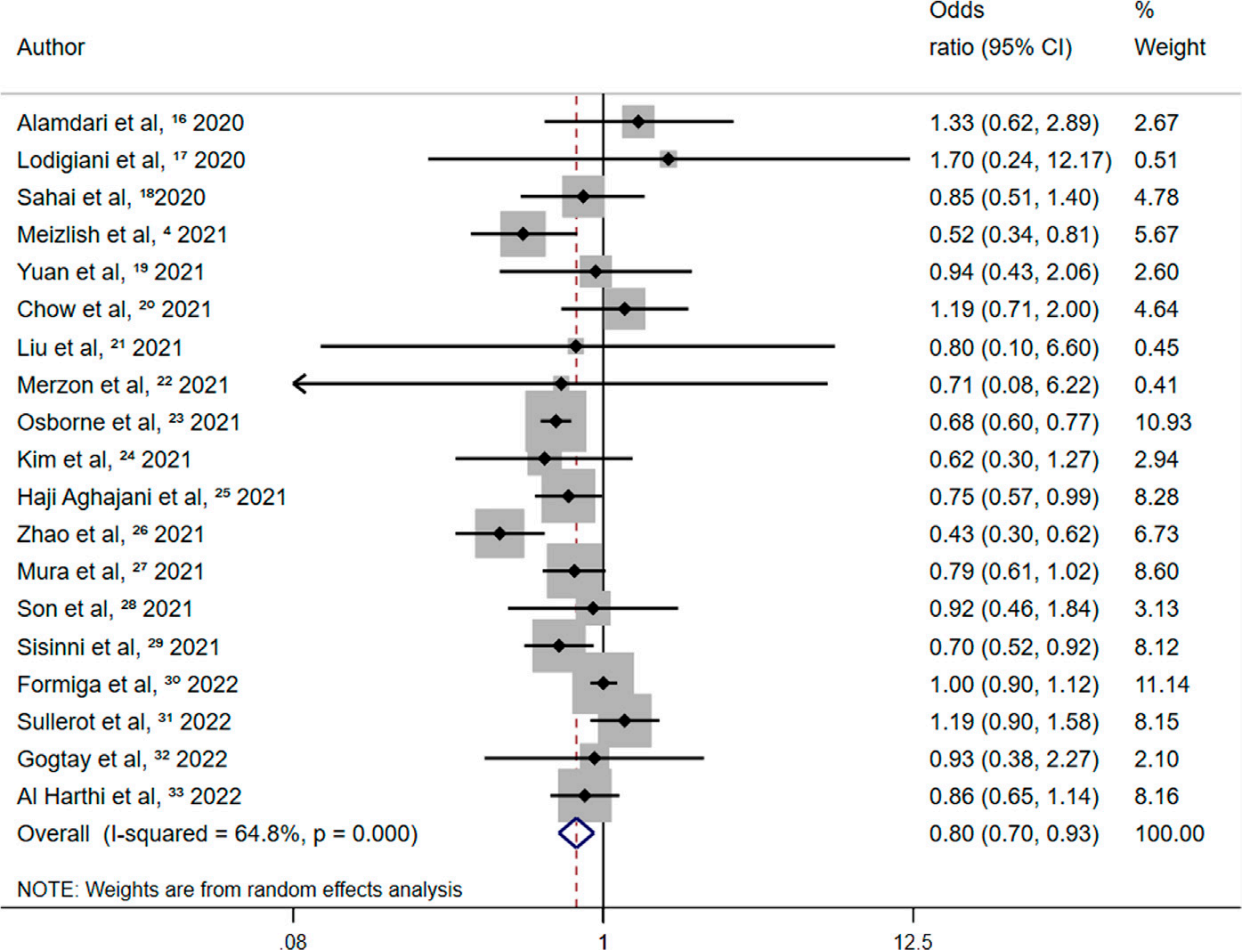
Summary odds ratio of all-cause mortality for aspirin use in retrospective studies.

Retrospective studies on the association between aspirin and all-cause mortality reported 2,152 deaths among 12,668 patients administered aspirin and 3,227 deaths among 27,645 patients in the control group. The summary random-effects OR was 0.80 (95% CI 0.70–0.93; *p* = 0.003) and high between-study heterogeneity was observed (I^2^ = 64.8%; *p* = 0.000) (Figure 4). A 20% reduction in all-cause mortality was observed after aspirin use among patients with COVID-19.

**FIGURE 5:**
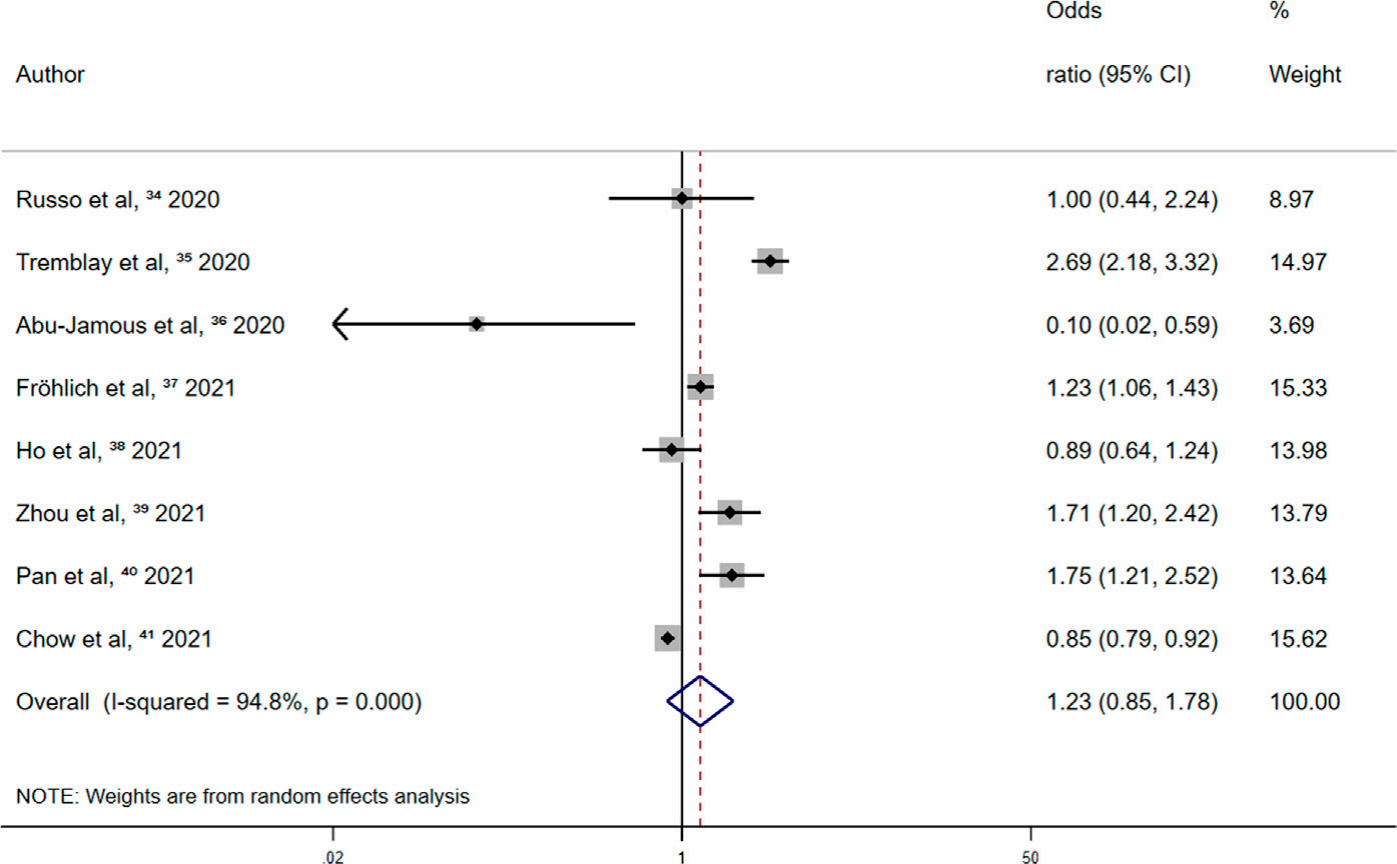
Summary odds ratio of all-cause mortality for other antiplatelet drugs use in retrospective studies.

Retrospective studies reported no significant effect between the use of other antiplatelet drugs and all-cause mortality among patients with COVID-19 [OR, 1.23 (95% CI 0.85–1.78; *p* = 0.283)] (Figure 5).

**FIGURE 6:**
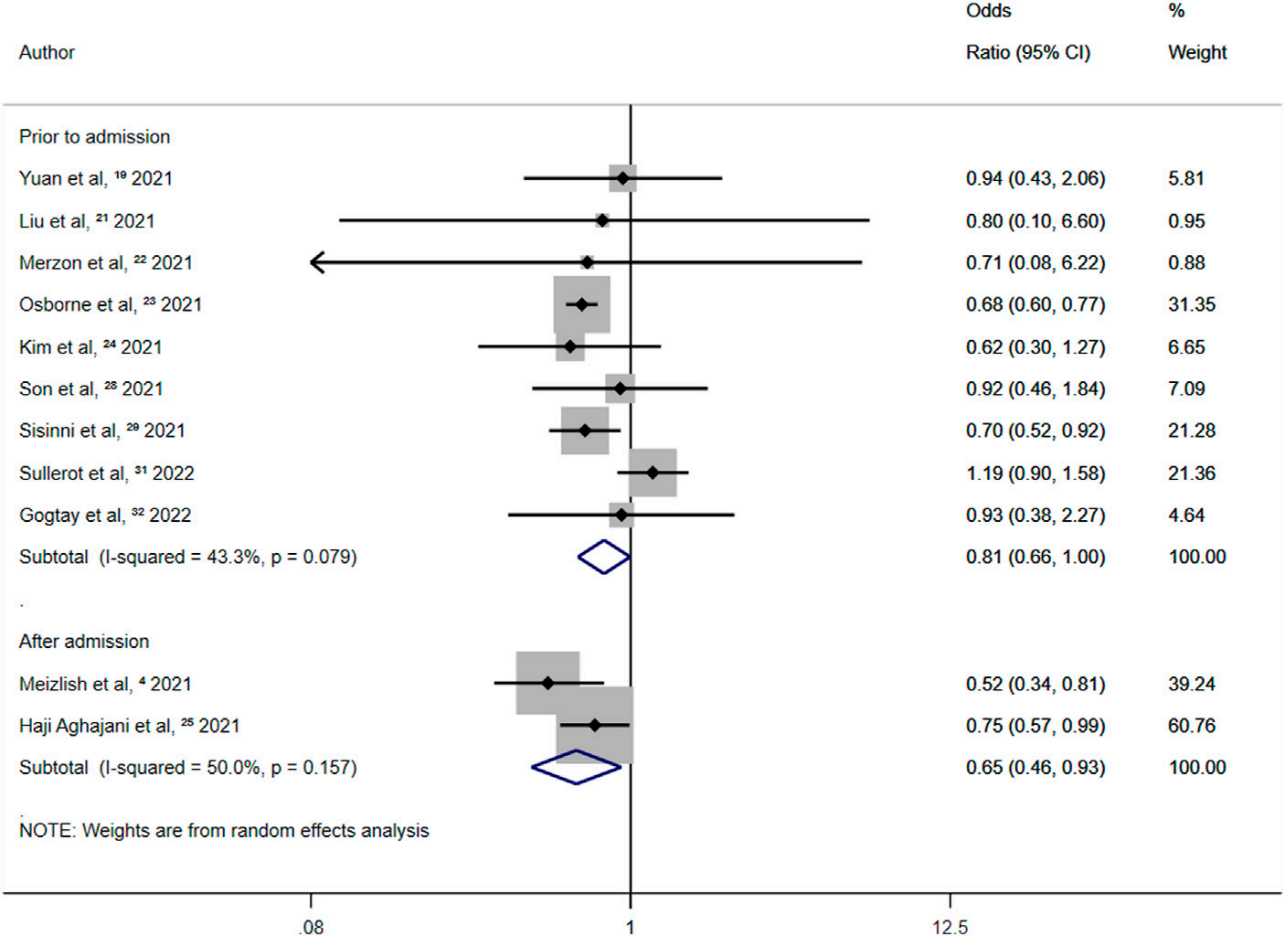
Summary odds ratio of all-cause mortality for aspirin use after admission and prior to admission.

In studies in which aspirin was administered after admission, the overall random-effects OR was 0.65 (95% CI 0.46–0.93; *p* = 0.018) (Figure 6). A 35% reduction in all-cause mortality among COVID-19 patients was observed for aspirin use after admission. In studies where aspirin was administered before admission, the overall random-effects OR was 0.81 (95% CI 0.66–1.00; *p* = 0.046) (Figure 6). Therefore, aspirin use before admission was independently associated with a 19% reduction in all-cause mortality in patients with COVID-19. The ratio of ORs was 1.25.

### 3.3 Associations between aspirin or other antiplatelet drug use and adverse events

**FIGURE 7:**
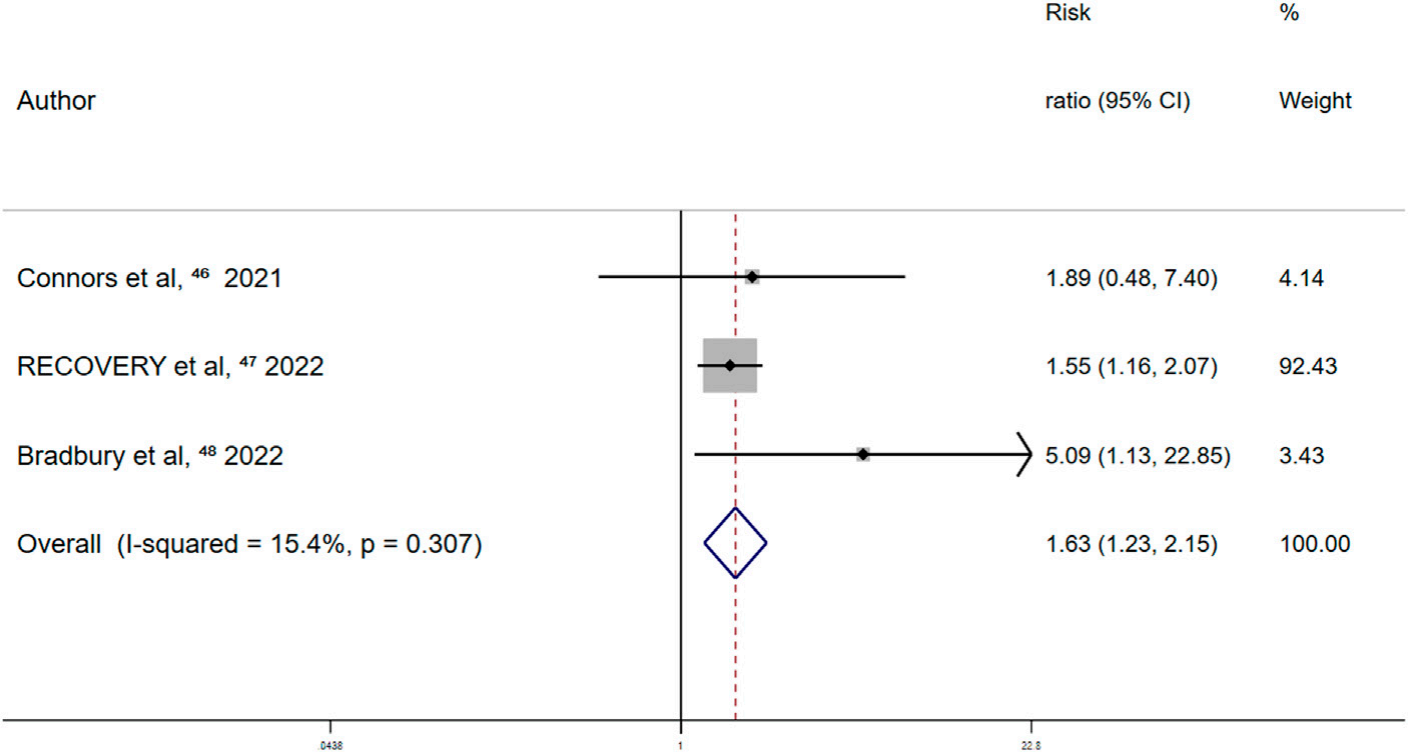
Summary hazard ratio of bleeding events for aspirin use in randomized controlled trials.

Randomized controlled trials on the association between aspirin use and major bleeding events reported a summary fixed-effects RR of 1.63 (95% CI 1.23–2.15; *p* = 0.001). Slight between-study heterogeneity was observed (I^2^ = 15.4%; *p* = 0.307) (Figure 7). Thus, aspirin use was independently associated with a 63% increased risk of major bleeding events in patients with COVID-19.

The randomized controlled trial on the association between P2Y12 inhibitors and major bleeding events reported an RR of 5.81 (95% CI 1.28–26.4). Thus, the use of P2Y12 inhibitors increased the risk of major bleeding events among patients with COVID-19.

**FIGURE 8:**
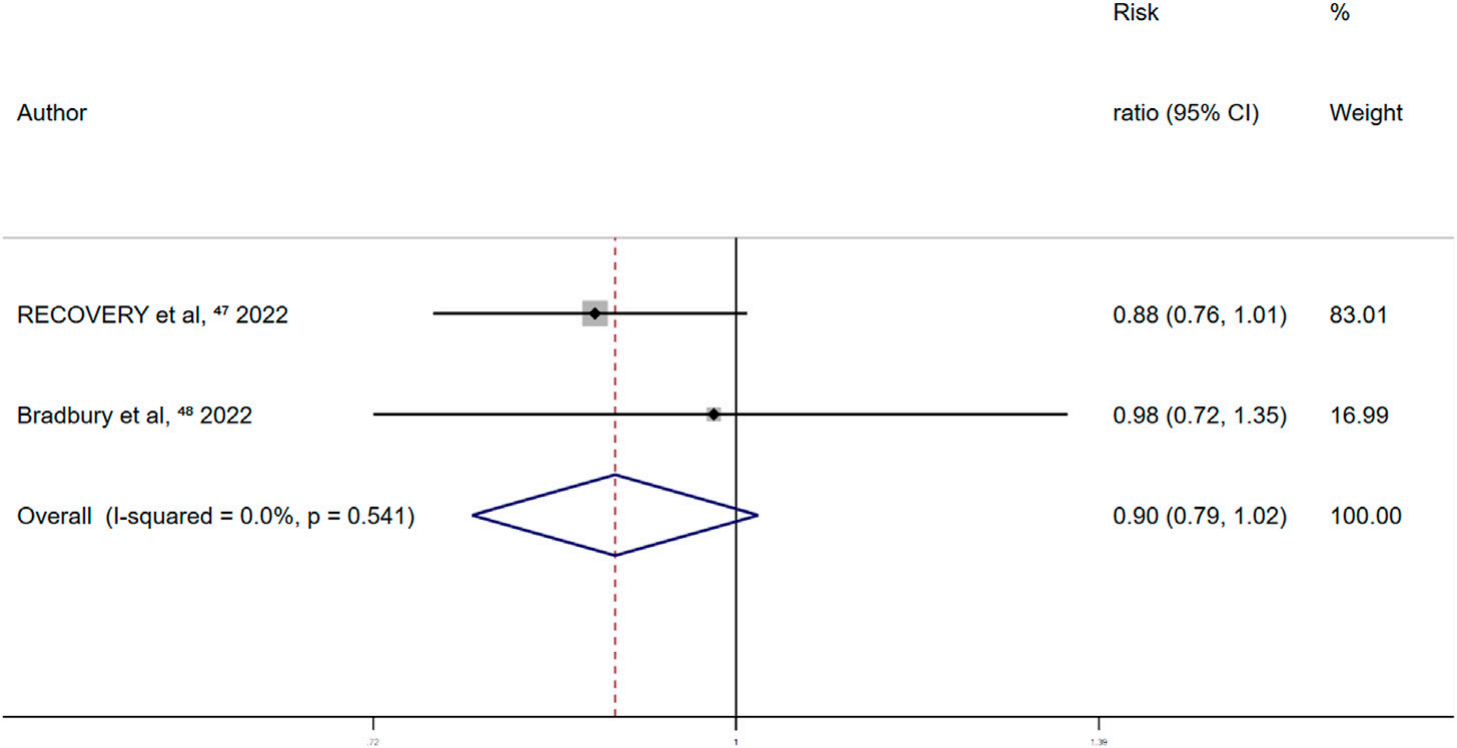
Summary hazard ratio of thrombotic events for aspirin use in randomized controlled trials.

Randomized controlled trials on the association between aspirin use and overt thrombosis events reported a summary fixed-effects RR of 0.90 (95% CI 0.79–1.02; *p* = 0.097). No between-study heterogeneity was observed (I^2^ = 0.0%; *p* = 0.541) (Figure 8). This indicated no significant effect of aspirin use on overt thrombosis events in patients with COVID-19.

The randomized controlled trial on the association between P2Y12 inhibitors and overt thrombosis events reported an RR of 0.77 (95% CI 0.53–1.11). This indicated no significant effect between the use of P2Y12 inhibitors and overt thrombosis events in patients with COVID-19.

**FIGURE 9:**
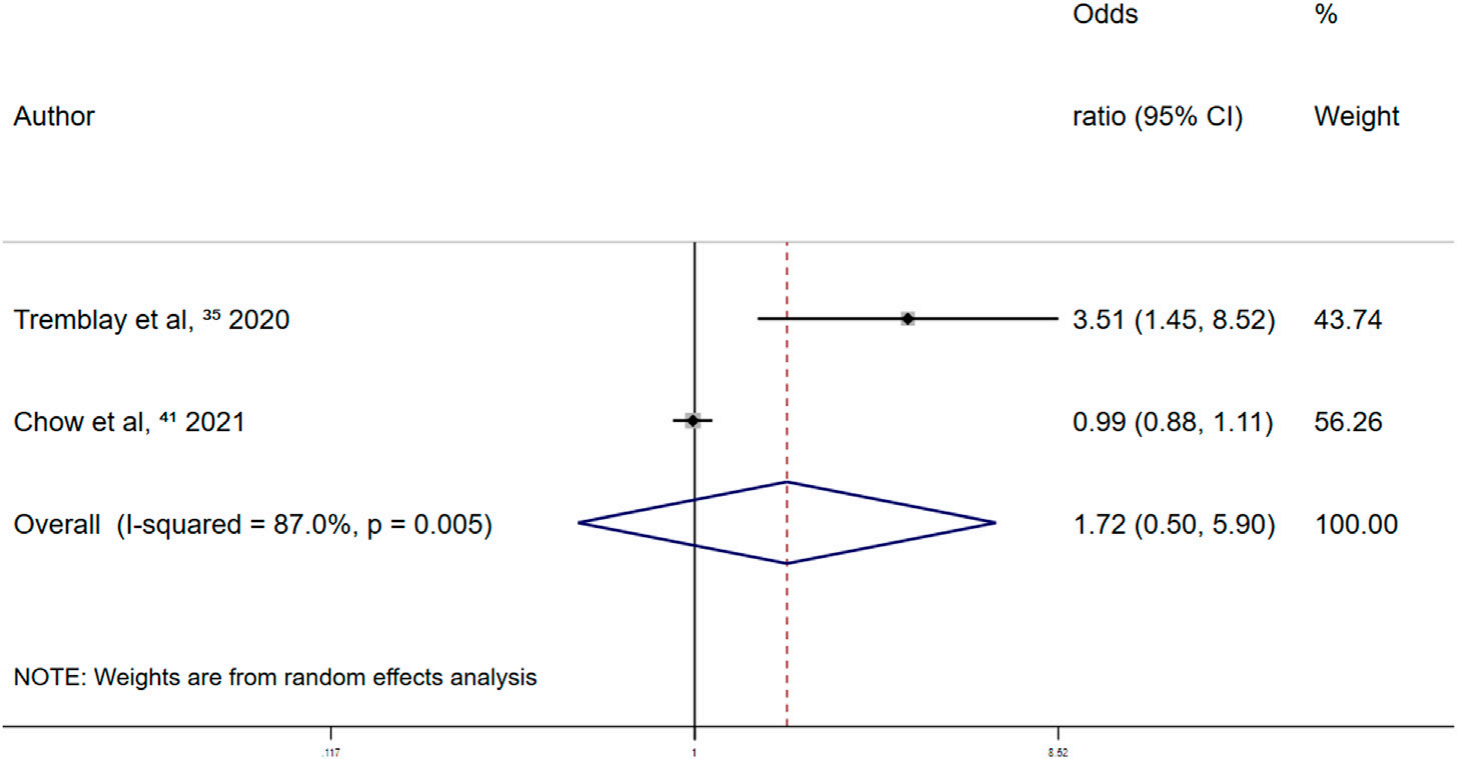
Summary odds ratio of bleeding events for other antiplatelet drugs use in retrospective studies.

Retrospective studies on the association between the use of other antiplatelet drugs and major bleeding events reported a summary random-effects OR of 1.72 (95% CI 0.50–5.90; *p* = 0.387) (Figure 9). This indicated that the use of other antiplatelet drugs was not significantly associated with major bleeding events in patients with COVID-19.

One retrospective study on the association between aspirin and major bleeding events reported an OR of 0.79 (95% CI 0.31–1.99). This indicated that the use of aspirin was not significantly associated with major bleeding events in patients with COVID-19.

One prospective cohort study on the association between aspirin and major bleeding events reported an OR of 1.14 (95% CI 1.07–1.22). Aspirin use resulted in a 14% increase in major bleeding events among patients with COVID-19.

One prospective cohort study that explored the association between other antiplatelet drugs and major bleeding events, reported an OR of 0.85 (95% CI 0.5–1.45). This indicated that the use of other antiplatelet drugs was not significantly associated with major bleeding events in patients with COVID-19.

**FIGURE 10:**
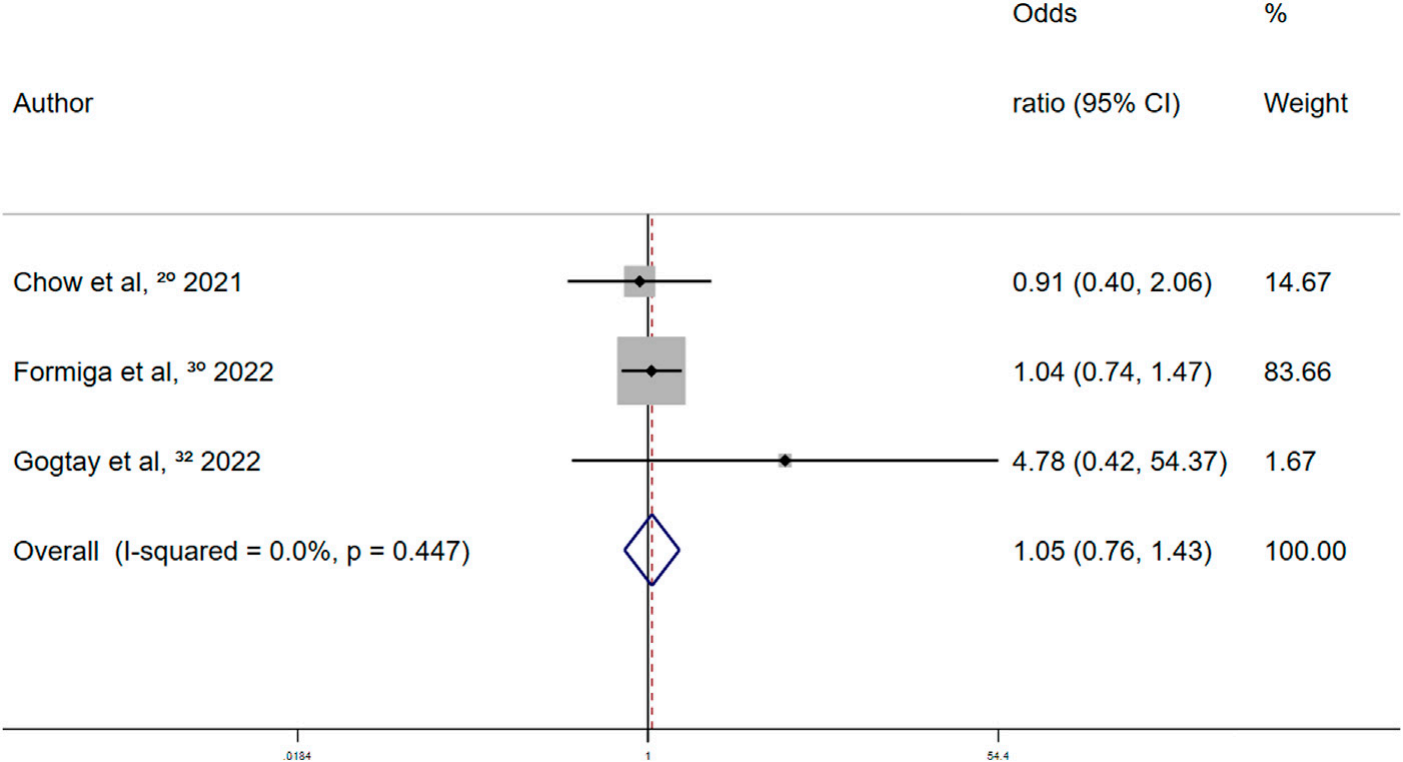
Summary odds ratio of thrombotic events for aspirin use in retrospective studies.

In the retrospective studies on the association between aspirin and overt thrombosis events, the summary fixed-effects OR was 1.05 (95% CI 0.76–1.43; *p* = 0.779) (Figure 10). Aspirin use and overt thrombosis events were not significantly associated in patients with COVID-19.

**FIGURE 11:**
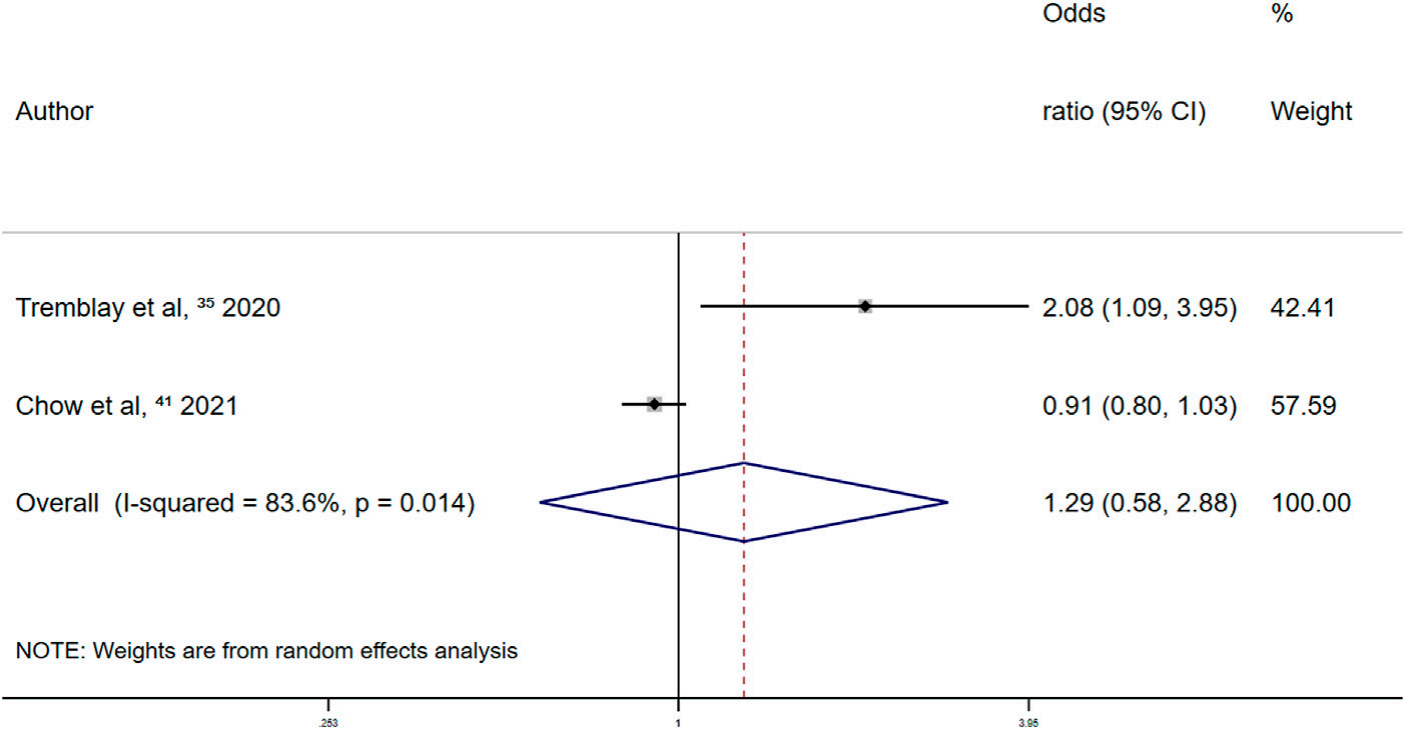
Summary odds ratio of thrombotic events for other antiplatelet drugs use in retrospective studies.

In the retrospective studies on the association between other antiplatelet drugs and overt thrombosis events, the summary random-effects OR was 1.29 (95% CI 0.58–2.88; *p* = 0.530) (Figure 11). Thus, the use of other antiplatelet drugs was not significantly associated with overt thrombosis events in patients with COVID-19.

One prospective cohort study on the association between aspirin and overt thrombosis events reported an OR of 0.55 (95% CI 0.47–0.64), corresponding to 45% fewer overt thrombosis events in patients with COVID-19 after aspirin use.

One prospective cohort study on the association between other antiplatelet drug use and overt thrombosis events reported an OR of 1.15 (95% CI 0.73–1.82). Hence, the use of other antiplatelet drugs was not significantly associated with overt thrombosis events in patients with COVID-19.

### 3.4 Quality assessment

The retrospective and prospective cohort studies showed low-to-moderate risks of bias based on the NOS. Table 1 shows the results of these quality assessments, indicating that of the retrospective and prospective cohort studies, 29 were good, while the remaining two were of fair quality. Regarding the three randomized controlled trials assessed by the Cochrane Risk of Bias Assessment Tool, the risk of bias was low in one trial in terms of mortality results and high in the other two trials, as these trials followed an open-label design (Table 2).

**TABLE 1:** The qualities of retrospective and prospective cohort studies assessed by the Newcastle-Ottawa scale.

	Selection	Comparability	Outcome	Total score	Quality of the study
Alamdari et al., 2020	***	*	***	7	Good
Lodigiani et al., 2020	**	**	***	7	Fair
Sahai et al., 2020	***	*	***	7	Good
Meizlish et al., 2021	***	**	***	8	Good
Yuan et al., 2021	**	**	***	7	Fair
Chow et al., 2021a	***	**	***	8	Good
Liu et al., 2021	***	**	***	8	Good
Merzon et al., 2021	***	*	***	7	Good
Osborne et al., 2021	***	*	***	7	Good
Kim et al., 2021	***	*	***	7	Good
Haji Aghajani et al., 2021	***	**	***	8	Good
Zhao et al., 2021	***	*	***	7	Good
Mura et al., 2021	***	*	***	7	Good
Son et al., 2021	***	**	***	8	Good
Sisinni et al., 2021	***	**	***	8	Good
Formiga et al., 2022	***	**	***	8	Good
Sullerot et al., 2022	***	**	***	8	Good
Gogtay et al., 2022	***	*	***	7	Good
Al Harthi et al., 2022	***	**	***	8	Good
Russo et al., 2020	***	*	***	7	Good
Tremblay et al., 2020	***	*	***	7	Good
Abu-Jamous et al., 2020	***	*	***	7	Good
Fröhlich et al., 2021	***	**	***	8	Good
Ho et al., 2021	***	**	***	8	Good
Zhou et al., 2021	***	*	***	7	Good
Pan et al., 2021	***	**	***	8	Good
Chow et al., 2021	***	**	***	8	Good
Chow et al., 2022	***	**	***	8	Good
Giacomelli et al., 2020	***	**	***	8	Good
Xiang et al., 2021	***	*	***	7	Good
Santoro et al., 2022	***	**	***	8	Good

*One criteria was followed; **Two criteria were followed; ***Three criteria were followed.

**TABLE 2:** The qualities of randomized controlled trials assessed by Cochrane Risk of Bias Assessment Tool.

	Connors et al., 2021	RECOVERY et al., 2022	Bradbury et al., 2022
Random sequence generation (selection bias)	low	low	low
Allocation concealment (selection bias)	low	low	low
Blinding of participants and personnel (performance bias)	low	high	high
Blinding of outcome assessment (detection bias)	low	high	high
Incomplete outcome data (attrition bias)	low	low	low
Selective reporting (reporting bias)	low	low	low
Other bias	low	low	low

## 4 Discussion

This meta-analysis included three randomized controlled trials comprising 17,225 patients, 27 retrospective studies including 96,552 patients, and four prospective cohort studies including 120,019 patients. The retrospective and prospective cohort studies showed that the administration of aspirin was associated with lower mortality among patients with COVID-19. Therefore, aspirin may be an effective treatment for COVID-19.

In one randomized controlled trial, other drugs may have affected the antithrombotic effect of aspirin. In the RECOVERY trial (RECOVERY Collaborative GroupAbbas et al., 2022), 94% and 93% of patients were taking corticosteroids and heparin, respectively, compared to 98% and 93% of patients in the study by Bradbury et al. (REMAP-CAP Writing Committee for the REMAP-CAP InvestigatorsBradbury et al., 2022) Both corticosteroids and heparin have antithrombotic effects, (Steinlin et al., 2017; Qiu et al., 2021), which may have prevented the exposed group from gaining significant benefits from aspirin.

The OR of the association between aspirin and all-cause mortality was lower when aspirin was administered after admission compared to that before admission. Thus, aspirin may be more beneficial in reducing all-cause mortality in patients with COVID-19 when administered after admission.

Traditionally, antithrombotic effects are achieved at low doses (75 mg/day–81 mg/day), analgesic and antipyretic effects are achieved at intermediate doses (650 mg–4 g/day), and anti-inflammatory effects are observed at high doses (4 g/day–8 g/day) of aspirin (Pillinger et al., 1998). Among the studies on aspirin dose included in the present meta-analysis, the aspirin dose was generally low to achieve an antithrombotic effect.

Evidence regarding aspirin reducing mortality in patients with COVID-19 is unclear. Previous studies mainly recognized the antiplatelet, antithrombotic, and anti-inflammatory effects of aspirin (Simon et al., 2020; Connors and Ridker, 2022). Aspirin is part of an effective antiplatelet regimen, (Antithrombotic Trialists' Collaboration, 2002), and exerts antithrombotic effects due to its inhibition of platelet function by the acetylation of platelet cyclooxygenase (COX)-1 at the functionally important amino acid residue serine 529 (Schrör, 1997). Our results showed that aspirin reduced mortality among patients with COVID-19, whereas other antiplatelet drugs did not, suggesting that the antiplatelet effects of aspirin may not reduce mortality among patients with COVID-19. However, the antiplatelet effects of aspirin may play a role in cases with bleeding, which is a serious adverse reaction.

The relationship between SARS-CoV-2 infection and the development of acute respiratory distress syndrome (ARDS) cannot be ignored (Asselah et al., 2021). SARS-CoV-2 infection can elevate plasma proinflammatory cytokine levels and cause an inflammatory “cytokine storm.” (Mehta et al., 2020; Ramasamy and Subbian, 2021) In sepsis and ARDS, multiple pathogenic mechanisms implicated in the development of multiple organ dysfunction can be modulated by aspirin (Toner et al., 2015; Yu et al., 2018). Regarding the pathogenesis and complications of COVID-19, inflammatory mediators are critical, among which aspirin has demonstrated a potential survival benefit through its anti-inflammatory actions in COVID-19 (Hussain et al., 2012; Kelleni, 2022). However, it is not clear whether antiplatelet therapy is associated with reduced morbidity in high-risk patients with ARDS (Wang et al., 2018).

The enzyme COX-2, which leads to the formation of prostaglandins, causes inflammation, swelling, pain, and fever (Schrör, 1997; Vane and Botting, 2003). However, aspirin and other non-steroidal anti-inflammatory drugs (NSAIDs) inhibit the activity of this enzyme to exert anti-inflammatory, analgesic, and antipyretic actions. NSAIDs do not significantly reduce the risk of SARS-COV-2 infection, (Moore et al., 2021), which may mean that aspirin does not reduce the mortality of patients with COVID-19 through its anti-inflammatory, analgesic, and antipyretic actions.

Aspirin has specific antiviral activities against influenza A and human rhinovirus (Glatthaar-Saalmüller et al., 2017). Published articles indicate the significant antiviral activities of aspirin against DNA and RNA viruses, including different human coronaviruses (Bianconi et al., 2020). Aspirin targeting of intracellular signaling pathways critical for viral replication could serve as a reliable adjunctive treatment option in patients with COVID-19 (Tantry et al., 2021).

Among deaths due to COVID-19, 11.8% of patients without underlying cardiovascular disease had severe heart damage, according to the National Health Commission (NHC) report (Zheng et al., 2020). Previous studies also reported that COVID-19 can cause myocardial damage and increase the incidence of cardiovascular diseases, resulting in increased mortality (Bianconi et al., 2020). The prognosis of patients with underlying cardiovascular disease but without myocardial injury is relatively favorable, (Clerkin et al., 2020; Guo et al., 2020), which highlights the correlation between COVID-19 and myocardial damage. However, various theories have been proposed regarding the mechanism by which COVID-19 causes an increased incidence of cardiovascular diseases. COVID-19 may cause the production of a variety of cytokines, resulting in coronary microvascular endothelial injury, plaque destruction, thrombosis generation and cardiomyocyte inflammation, apoptosis, and necrosis (Guo et al., 2020; Zheng et al., 2020). SARS-CoV-2 hydrolyzes the S protein through serine protease, binds with transmembrane angiotensin-converting enzyme 2 (ACE2), and enters cardiomyocytes, which may lead to viral myocarditis (Bonow et al., 2020; Zheng et al., 2020). The reduction of COVID-19 mortality due to aspirin use may be attributed to the reduction of myocardial injury by aspirin’s anti-inflammatory and antithrombotic effects, as well as its protective effects in the coronary artery. However, the outcome of the 34 studies included in this analysis was all-cause rather than cardiovascular disease mortality. Therefore, whether aspirin reduces the mortality of patients with COVID-19 owing to its protective effect on the coronary artery and myocardium requires further research.

**FIGURE 12:**
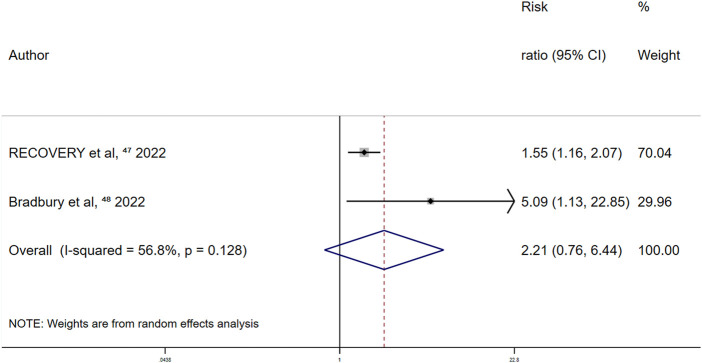
Summary hazard ratio of bleeding events for heprin use in randomized controlled trials.

Prospective cohort studies showed that aspirin increases the risk of bleeding events and decreases the risk of thrombotic events. The three randomized controlled trials assessed bleeding events and concluded that aspirin was associated with higher risks of bleeding events. In the randomized controlled trials, the random-effects RR for the trials that did and did not use heparin were 2.21 (95% CI 0.76–6.44) (Figure 12) and 1.89 (95% CI 0.48–7.4), respectively, suggesting an increased risk of bleeding with heparin use. Two randomized controlled trials mentioned thrombotic events, in which aspirin did not reduce the risk of thrombotic events in patients with COVID-19.

### Limitations

This study had several limitations. First, retrospective and prospective cohort studies accounted for a large proportion (92.6%) of the studies included in the meta-analyses, which may be prone to bias. Second, owing to the observational nature of the present study, selection bias was possible, as patients requiring aspirin may be at high risk for potential comorbidities or thrombosis. Third, the data were only searched in PubMed, which may have missed relevant articles. Fourth, our study only included an assessment of mortality over a relatively short period and did not include reports of long-term mortality, including post-discharge mortality. Fifth, the trials recruited only adults; thus, the effect of aspirin on children remains unclear. Sixth, the endpoint difference in mortality may have led to inconsistencies among trial results. Seventh, some ongoing randomized controlled trials were not included in our study. Eighth, in the retrospective studies, the dosage of aspirin was mainly the conventional dose, and high doses were ignored, which may have led to incomplete results. Ninth, among randomized controlled trials, the sample size of the RECOVERY study was large, with a weight of 86.47%, (RECOVERY Collaborative GroupAbbas et al., 2022), which may have caused bias. Tenth, studies on the effect of anticoagulants were excluded, (Viecca et al., 2020; Matli et al., 2021; Rivera-Caravaca et al., 2021; Berger et al., 2022; Santoro et al., 2022b; Rivera-Caravaca et al., 2022), which could lead to bias. For example, although approximately 10% of the patients in these two studies were taking antiplatelet drugs, the main purpose aimed at determining the effect of oral anticoagulants on the prognosis of patients with COVID-19; thus, some studies were excluded (Rivera-Caravaca et al., 2021; Rivera-Caravaca et al., 2022). More recently, Santoro et al. demonstrated the association of aspirin combined with prophylactic anticoagulation therapy with lower mortality risk among patients with COVID-19 (Santoro et al., 2022b). Eleventh, the prognosis of patients with COVID-19 is influenced by many factors, such as cancer, (Pérez-Segura et al., 2021), arterial hypertension, (El-Battrawy et al., 2021), and kidney disease, (Uribarri et al., 2020), which may bias the results of this study, as not all 34 studies were well matched with these factors.

## 5 Conclusion

The results of this meta-analysis of patients with COVID-19 showed that the administration of aspirin may reduce all-cause mortality; however, these findings require confirmation by randomized controlled trials.

## Data Availability

The original contributions presented in the study are included in the article/Supplementary Material, further inquiries can be directed to the corresponding authors.
